# Effect of probiotic root canal irrigation on macrophage polarization in rat periapical lesions

**DOI:** 10.1590/1807-3107bor-2026.vol40.082

**Published:** 2026-10-23

**Authors:** Gabriela Leite Borges, Ana Paula Gomes-Moura, Lisa Danielly Curcino Araujo, Aluísio Eustáquio Miranda-Filho, Marília Pacífico Lucisano, Sérgio Luiz Salvador, Flávia Aparecida Chaves Furlaneto, Michel Reis Messora, Paulo Nelson-Filho, Raquel Assed Bezerra da Silva, Léa Assed Bezerra da Silva

**Affiliations:** (a) Universidade de São Paulo – USP, School of Dentistry of Ribeirão Preto, Ribeirão Preto, SP, Brasil.; (b) Universidade de São Paulo – USP, School of Dentistry of Ribeirão Preto, Department of Pediatric Dentistry, Ribeirão Preto, SP, Brasil.; (c) Universidade de São Paulo – USP, School of Pharmaceutical Sciences of Ribeirão Preto, Department of Clinical Analyses, Toxicology and Food Science, Ribeirão Preto, SP, Brasil.; (d) Universidade de São Paulo – USP, School of Dentistry of Ribeirão Preto, Department of Oral and Maxillofacial Surgery, Oral Medicine and Periodontology, Ribeirão Preto, SP, Brasil.

**Keywords:** Probiotics, Periapical lesion, Immunohistochemistry, Macrophage polarization

## Abstract

The aim of this study was to evaluate the effect of the probiotic *Bifidobacterium animalis* subsp. *lactis* HN019 as a root canal irrigant on the polarization of macrophages into M1 and M2 subtypes in periapical lesions induced in rats, using immunohistochemical analysis. Forty-eight adult male Wistar Hannover rats were divided into six groups: Group I (4 animals/8 teeth): sound teeth; Group II (4 animals/4 teeth): 2.5% sodium hypochlorite as the irrigation solution (3 days); Group III (4 animals/4 teeth): probiotic solution as the irrigant (3 days); Group IV (12 animals/24 teeth per period): periapical lesions without irrigation (7, 21, and 42 days); Group V (12 animals/24 teeth per period): 2.5% sodium hypochlorite irrigation (7, 21, and 42 days); Group VI (12 animals/24 teeth per period): probiotic irrigation (7, 21, and 42 days). The animals were euthanized following the experimental periods. Immunohistochemical analysis was performed using NOS2 and CD163 markers for the M1 and M2 subtypes, respectively. The findings showed no significant differences in NOS2 and CD163 expression across the experimental periods (3, 7, 21, and 42 days) or between the irrigated groups, except at 42 days, when NOS2 expression was higher in the probiotic-treated group than in the control group. The probiotic *Bifidobacterium animalis* subsp. *lactis* HN019 demonstrated the potential to modulate the immune-inflammatory response in periapical lesions by increasing NOS2 expression 42 days after pulp exposure.

## Introduction

Macrophages play a crucial role in the inflammatory response of periapical tissue in response to bacterial infection within the root canal system, capable of polarizing into distinct functional subtypes.^1,2^ The M1 subtype is associated with a pro-inflammatory response, while the M2 subtype is related to anti-inflammatory responses and tissue repair processes.^3,4^ Recent research has highlighted the effects of various probiotic strains on the polarization of macrophages into M1 and M2 subtypes in systemic diseases, underscoring their importance in controlling associated inflammation.^5–7^ In the context of oral diseases, it has been shown that *in vitro* stimulation of macrophages by different endodontic cements can alter the phenotypic polarization of these cells.^4^ Additionally, M1 and M2 macrophage subtypes have been identified in biopsies of radicular cysts from both primary and permanent human teeth, with a predominance of M2 cells.^7^

Endodontic treatment aims to eliminate pathogenic microorganisms and promote the resolution of inflammation.^4,8^ However, conventional irrigant solutions, such as sodium hypochlorite and chlorhexidine, may have limitations in effectively eliminating bacteria and modulating the inflammatory process.^9–11^ Consequently, there is a growing interest in antimicrobial substances that can act within root canals without causing harm to soft tissues and the patient.^12,13^

In this context, the therapeutic use of probiotics has significantly increased in both the medical and dental fields in recent years.^14–16^ These beneficial microorganisms are capable of modulating the immune-inflammatory response and have demonstrated efficacy in preventing and treating various diseases, including inflammatory disorders.^17,18^ In dentistry, research has particularly explored probiotics as an effective therapeutic alternative for managing chronic processes such as periodontitis, with promising findings in periodontal therapy.^19,20^

In Endodontics, some studies have also indicated beneficial effects of probiotic strains, such as *Lactobacillus rhamnosus* and *Lactobacillus acidophilus*, in treating periapical lesions.^21,22^ Additionally, the probiotic *Bifidobacterium animalis* subsp. *lactis* HN019 has been shown to exhibit therapeutic potential against the main microorganisms present in root canal infections, with demonstrated antimicrobial activity *in vitro*, as well as promising effects and possible mechanisms of action in both health and various pathological conditions.^23^ More recently, probiotic-based irrigation has also been reported to modulate host innate immune responses in periapical lesions, including the reduction of neutrophil extracellular trap formation and the stimulation of antimicrobial peptide CRAMP (LL-37 in humans) expression.^24^

Taken those results together, evidence indicating that probiotic-based irrigation can influence the host immune response in periapical lesions, not only through antimicrobial effects but also by modulating neutrophil-mediated mechanisms and antimicrobial peptide expression, highlights the need to further elucidate the biological pathways involved in their therapeutic action. Therefore, advancing knowledge on the role of probiotics in macrophage polarization becomes particularly relevant for understanding their impact on the inflammatory microenvironment and tissue repair.

The null hypothesis of this study was that the use of *Bifidobacterium animalis* subsp. *lactis* HN019 as a root canal irrigant would not influence macrophage polarization (M1/M2) or the immune-inflammatory response in experimentally induced periapical lesions in rats when compared with conventional irrigation or no irrigation.

## Methods

### Animal ethics and welfare

This manuscript has been written according to the Preferred Reporting Items for Animal Studies in Endodontology (PRIASE) 2021 guidelines^25^ (Figure 1) and the ARRIVE guidelines for animal studies.^26,27^ It was approved by the Ethics Committee on Animal Use of the Ribeirão Preto School of Dentistry, University of São Paulo (FORP-USP), Ribeirão Preto, Brazil (CEUA Protocol No. 2018.1.782.58.0). Male rats, weighing approximately 170-250 grams, were obtained at 7 weeks of age. After a one-week acclimation period, they were housed in polypropylene cages at the Animal Facility I of the Ribeirão Preto School of Dentistry, University of São Paulo (FORP-USP), Ribeirão Preto, Brazil.. The ambient temperature was maintained at 22°C (±2°C) with a 12-hour light-dark cycle, and animals had free access to standard rodent feed and water.

**Figure 1:**
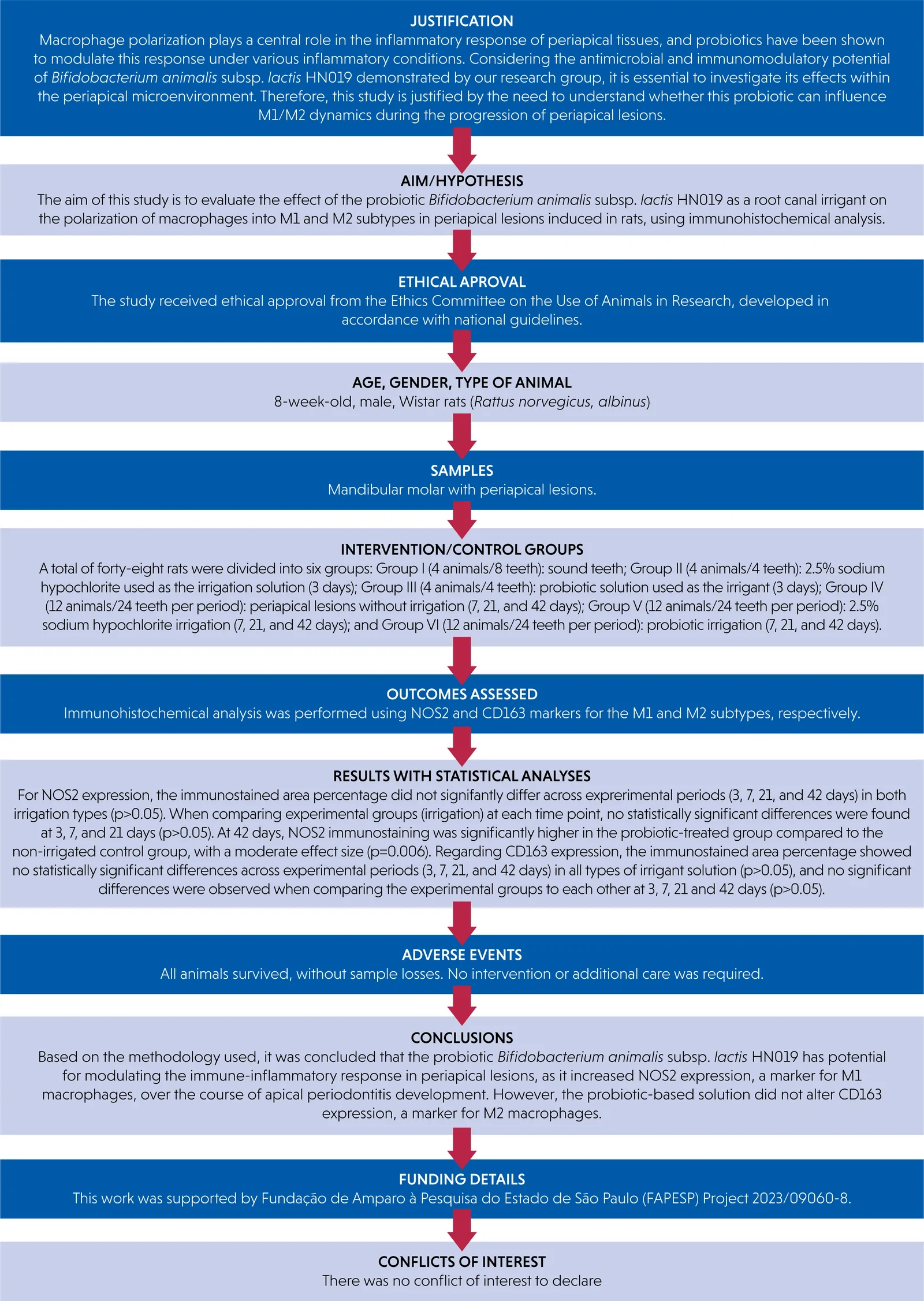
Flowchart from Preferred Reporting Items for Animal Studies in Endodontology (PRIASE 2021).

### Sample Size

Based on a previous study,^23^ the sample size was determined to ensure a statistical power of 80% for detecting significant differences of 20% between groups. Considering an alpha error of 5%, a minimum of seven animals per group was deemed necessary. Taking into account potential losses during sample collection and processing, eight animals per group were ultimately used.

### Study design

Forty-eight adult male Wistar Hannover rats (*Rattus norvegicus albinus*) were divided into six groups according to the irrigant solution, as follows: Group I (4 animals/8 teeth), sound teeth; Group II (4 animals/4 teeth), 2.5% sodium hypochlorite as the irrigant solution (3 days); Group III (4 animals/4 teeth), probiotic as the irrigant solution (3 days); Group IV (12 animals/24 teeth per period), periapical lesion without irrigation (7, 21, and 42 days); Group V (12 animals/24 teeth per period), 2.5% sodium hypochlorite as the irrigant solution (7, 21, and 42 days); Group VI (12 animals/24 teeth per period): probiotic as the irrigant solution (7, 21, and 42 days).

The tooth was considered the experimental unit in this study, given the localized nature of the endodontic intervention and outcome assessment. Each tooth was treated as an independent sample according to the assigned experimental condition. To reduce potential intra-animal bias, treatments were systematically distributed between the right and left hemimandibles, ensuring controlled and balanced allocation within each animal.

### Preparation of probiotic irrigation solution

The preparation of the probiotic-based irrigant solution was based on a previously published study.^23^ The probiotic *Bifidobacterium animalis* subsp. *lactis* HN019 (HOWARU Bifido, E.I. Dupont^®^ de Nemours and Company, Wilmington, DE, USA) was cultured on MRS agar medium (Man, Rogosa and Sharpe – D™ Lactobacilli MRS Broth, Sparks, MD, USA) for 48 hours at 37 °C under anaerobic conditions (GASPAK™ EZ Anaerobe Container System with indicator, Sparks, MD, USA). Subsequently, using a sterile inoculating loop, the bacterial inoculum was transferred to Falcon centrifuge tubes containing glass beads and sterile distilled water. After homogenization in a tube shaker (Phoenix AP 65, Araraquara, SP, Brazil), the optical density (OD) was determined at a wavelength of 625 nm using a spectrophotometer (Micronal AJX-1000, São Paulo, SP, Brazil). The suspension was then subjected to serial decimal dilution up to 10^–9^ in phosphate-buffered saline (PBS) solution (pH 7.0). Diluted aliquots of 50 μL were plated onto Petri dishes containing MRS agar and spread using a sterile angled glass rod. Following inoculation, the plates were incubated under anaerobic conditions for 48 hours at 37°C. After the incubation period, colony-forming units (CFU) per mL were counted in duplicate. An OD of 2.007 was obtained, corresponding to a standardized inoculum of 2.7 × 10^9^ CFU/mL (Oliveira et al., 2017). The root canal irrigating solution was prepared by adding 100 μL of the suspension containing 2.7 × 10^9^ CFU/mL of *B. animalis* subsp. *lactis* HN019 to 2.0% carboxymethylcellulose.

### Induction of periapical lesions

Initially, the animals were anesthetized by intraperitoneal injection of xylazine (Rompum^®^, Bayer Animal Health, São Paulo, SP, Brazil; 10 mg/kg body weight) and ketamine (Dopalen^®^, Agribrands Purina do Brasil Ltda., Paulínia, SP, Brazil; 80 mg/kg body weight). Subsequently, the animals from the groups in which periapical lesions were induced were immobilized and positioned on a mandibular retraction table, remaining with their mouths open to allow adequate visualization of the mandibular molars. Coronal access was performed using a spherical bur (No. 1/2 – KG Sorensen, Cotia, SP, Brazil) mounted on a low-speed handpiece and contra-angle (Dabi Atlante Equipamentos Odontológicos, Ribeirão Preto, SP, Brazil) until the entrance to the root canals was visualized and confirmed with a size 10 K-type endodontic file (Maillefer S/A, Ballaigues, Switzerland) directed toward the distal root.

The pulp chamber was initially left exposed to the oral environment to allow contamination of the root canals and subsequent development of periapical lesions. After 3 days, Groups I, II, and III were euthanized. In Group IV, after pulp chamber exposure, an endodontic file was inserted to verify canal patency, and the canals were maintained open for 7, 21, and 42 days to evaluate lesion progression without any treatment (negative control). In Groups V and VI, after the irrigation procedures, the teeth were sealed with silver amalgam (Vallory^®^, SS White Artigos Dentários Ltda., Rio de Janeiro, RJ, Brazil) to prevent extravasation of the irrigant material. Following the experimental periods, the animals were anesthetized and euthanized with a lethal dose of isoflurane (Isoforine^®^, Cristália Produtos Químicos Farmacêuticos Ltda., Itapira, SP, Brazil).

### Histological processing

After euthanasia, the hemimandibles were removed using sterile surgical scissors and fixed by immersion in 10% buffered formalin until the demineralization process. For specimen demineralization, a 4.13% EDTA-based solution (pH 7.0–7.4) was used. The specimens were maintained in this solution at room temperature, with weekly solution changes until complete demineralization was achieved. Following demineralization, the specimens were subjected to routine histotechnical processing. They were washed under running water for 2 hours, dehydrated in ascending concentrations of ethanol (70% and 95% for 30 minutes each; two changes of 100% ethanol for 20 minutes each; and two additional changes of 100% ethanol for 40 minutes each), cleared in xylene (two baths of 20 minutes and one of 40 minutes), and embedded in paraffin.

The paraffin blocks containing the teeth were longitudinally sectioned in a mesiodistal direction using a microtome (Leica RM2145; Leica Microsystems GmbH, Wetzlar, Germany). Semi-serial sections of 5 μm thickness were obtained at 15 μm intervals throughout the entire extent of the periapical lesion, except in the apical foramen region, where all sections were collected without intervals. For each sample, 15 to 25 slides were obtained, with three sections mounted on each slide.

### Immunohistochemical analysis

#### Immunostaining for NOS2 (M1 Subtype)

Sequential histological sections were deparaffinized, hydrated, and rinsed. Antigen retrieval was conducted by immersing slides in citrate buffer (pH=6.0), and endogenous peroxidase was blocked using a 3% hydrogen peroxide solution for 30 minutes. After washing in PBS, nonspecific binding was blocked with a 5% BSA/PBS solution for 1 hour. The primary antibody for M1 immunostaining (NOS2, 1:500, a00368-1, Boster Biological Technology, USA) was incubated overnight at 4°C. After a PBS wash, the secondary antibody (1:200, anti- rabbit, sc 2491, Santa Cruz Biotechnology, Brazil) was incubated for 1 hour. Following an additional PBS wash, sections were incubated with an avidin-biotin-peroxidase complex (ABC kit, Vecstain; Vector Laboratories Inc.) for 30 minutes. After a final PBS wash, staining was developed with diaminobenzidine (DAB; Sigma-Aldrich Corporation, Saint Louis, USA). Slides were then washed with distilled water and counterstained with Harris hematoxylin for 10 seconds. Following a 30-second wash with ammoniacal water and another rinse in running water, the slides were cleared, dehydrated, and mounted with Entellan (Merck, Darmstadt, Germany) and coverslips.

#### Immunostaining for CD163 (M2 Subtype)

Sequential histological sections were deparaffinized, hydrated, and rinsed. Antigen retrieval was achieved by immersing slides in buffer (pH=9). Subsequently, slides were rinsed five times in distilled water, followed by a wash in DAKO EnVision™ Kit Wash Buffer (code K8002; Dako/Agilent Technologies, Carpinteria, CA, USA) Endogenous peroxidase blocking was performed with a peroxidase blocking solution for 10 minutes. Slides were then washed with wash buffer for 5 minutes, and nonspecific binding was blocked with a 1% BSA/PBS solution for 30 minutes. The primary antibody for M2 immunostaining (CD163, 1:10, sc 58965, Santa Cruz Biotechnology, Brazil) was incubated overnight at 4°C. After a 5-minute wash with wash buffer, slides were incubated with Envision Flex Linker (mouse) for 15 minutes. Following a subsequent wash, sections were incubated with Envision Flex/HRP for 20 minutes. The slides were washed again with wash buffer, and staining was developed with EnVision™ FLEX Substrate Working Solution (DAB) (Dako/Agilent Technologies, Carpinteria, CA, USA). Slides were again rinsed in wash buffer for 5 minutes and counterstained with Harris hematoxylin for 10 seconds. After three rinses in running water, the slides were dehydrated, cleared, and mounted with Entellan (Merck, Darmstadt, Germany) and coverslips.

The analysis area was standardized to the apical and periapical regions. Histological images were obtained using a 20× objective with a light microscope (Axio Imager.M1, Carl Zeiss MicroImaging GmbH, Göttingen, Germany). For all specimens, the field of view was centered on the apical foramen and included the largest possible extent of the periapical lesion, ensuring consistent field selection across samples. Digital images were captured under standardized magnification and illumination conditions and subsequently analyzed using ImageJ software (National Institutes of Health, Bethesda, MD, USA). The software was used to quantify the percentage of positively stained area (brown color) within the total field area of each image, and the results were expressed as the percentage of stained area per analyzed field.^24^

### Statistical analysis

Comparisons between groups were conducted at a 5% significance level (α=0.05) using the JAMOVI statistical package (version 2.5.4, Sydney, Australia), with outcomes showing p-values <0.05 considered statistically significant. Comparisons across time points within the same group were performed using the Kruskal-Walli's test (H), followed by the Dwass-Steel-Critchlow-Fligner post hoc test, which accounts for multiple comparisons. Comparisons between irrigation types within the same experimental period were conducted using the Mann-Whitney test, with the point-biserial rank correlation coefficient used as a measure of effect size.

## Results

Table presents the statistical analysis comparing the immunostained area percentage for NOS2 and CD163 among groups and experimental periods. For NOS2 expression, the immunostained area percentage did not significantly differ across experimental periods (3, 7, 21, and 42 days) in both irrigation types (p>0.05). When comparing experimental groups (irrigation) at each time point, no statistically significant differences were found at 3, 7, and 21 days (p>0.05). At 42 days, NOS2 immunostaining was significantly higher in the probiotic-treated group compared to the non-irrigated control group, with a moderate effect size (p=0.006). Regarding CD163 expression, the immunostained area percentage showed no statistically significant differences across experimental periods (3, 7, 21, and 42 days) in all types of irrigant solution (p>0.05), and no significant differences were observed when comparing the experimental groups to each other at 3, 7, 21, and 42 days (p>0.05).

**Table 1:** Statistical analysis of immunostained area percentage (%) for NOS2 and CD163 among groups and experimental periods (median [IQR]).

Irrigant/Period		3 days	7 days	21 days	42 days	p-value I
		Percent (%)				
NOS2						
	No Irrigation	4.01 (1.33)^aA^	4.62 (1.13)^aA^	3.60 (0.51)^aA^	2.86 (1.22)^aB^	0.395
	Sodium Hypochlorite	4.43 (3.18)^aA^	6.40 (3.65)^aA^	6.13 (3.43)^aA^	6.28 (4.60)^aBC^	0.570
	Probiotic Solution	5.50 (3.31)^aA^	7.02 (1.96)^aA^	5.54 (3.79)^aA^	10.2 (5.39)^aC^	0.176
	p-value II	0.794	0.206	0.411	0.006* (0.563)	
CD163						
	No Irrigation	2.61 (2.30)^aA^	3.53 (1.59)^aA^	3.91 (5.03)^aA^	4.45 (1.77)^aA^	0.392
	Sodium Hypochlorite	3.89 (1.24)^aA^	4.28 (3.55)^aA^	3.94 (3.25)^aA^	4.59 (4.83)^aA^	0.983
	Probiotic Solution	6.53 (2.21)^aA^	5.67 (0.64)^aA^	5.88 (1.68)^aA^	4.98 (0.89)^aA^	0.653
	p-value II	0.119	0.176	0.575	0.992	

p < 0.05 indicates statistical significance. Values are presented as median (interquartile range [IQR]). p-value I: Comparison among experimental periods (3, 7, 21, and 42 days) within the same group, using the Kruskal-Wallis test followed by the Dwass-Steel-Critchlow-Fligner post hoc test (ε² as a measure of effect size). Different uppercase letters indicate statistically significant differences among periods within the same group. p-value II: Comparison among irrigation protocols (no irrigation, sodium hypochlorite, and probiotic solution) within the same experimental period, using the Mann-Whitney test (biserial rank correlation as a measure of effect size). Lowercase letters indicate comparisons across values within the same row (horizontal), whereas uppercase letters indicate comparisons among values within the same column (vertical).

Figures 2 and 3 illustrate the microscopic findings following the immunohistochemical assay for NOS2 and CD163 antibodies.

**Figure 2:**
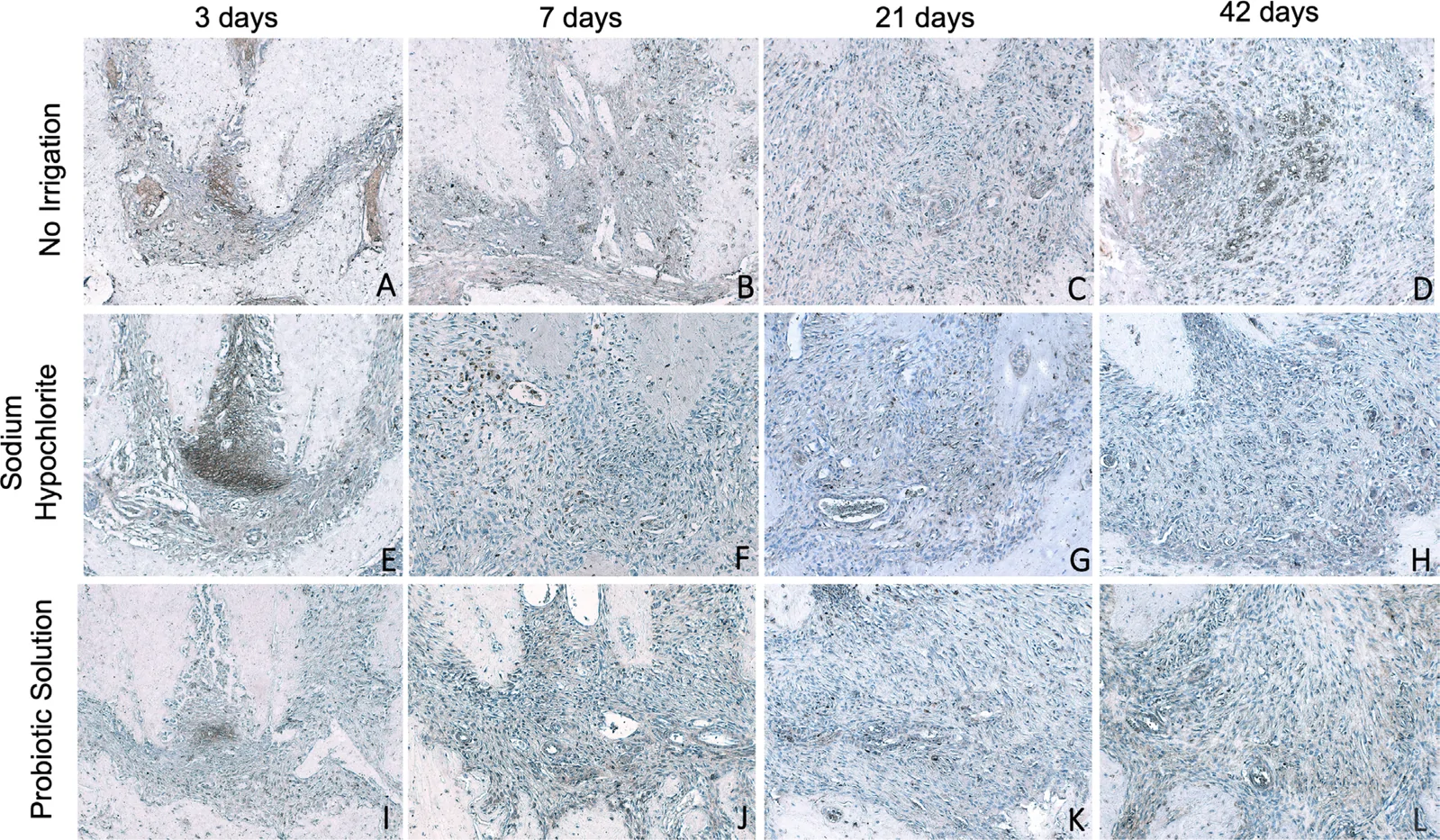
Representative photomicrographs of immunostaining for NOS2 (a marker indicative of M1 macrophages) throughout the development of the periapical lesion (3, 7, 21, and 42 days), in both the non-irrigation groups and the groups that received irrigation with 2.5% sodium hypochlorite and probiotic (Heiss, 20x).

**Figure 3:**
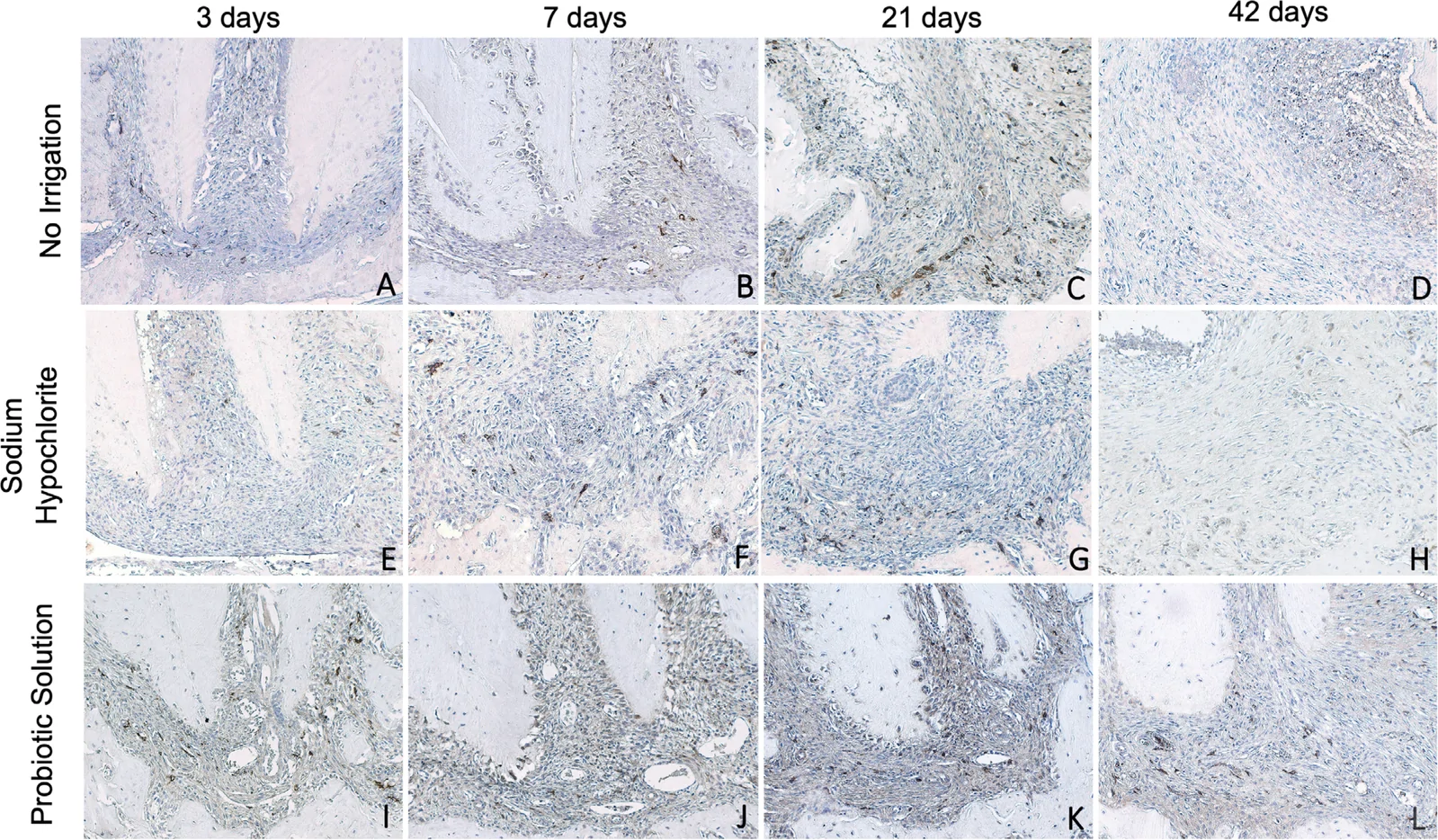
Representative photomicrographs of immunostaining for CD163 (a marker indicative of M2 macrophages) throughout the development of the periapical lesion (3, 7, 21, and 42 days), in both the non-irrigation groups and the groups that received irrigation with 2.5% sodium hypochlorite and probiotic (Heiss, 20x).

## Discussion

During the inflammatory response, macrophages polarize into subtypes such as M1 and M2, which have distinct phenotypic characteristics.^1–4^ Simultaneously, the therapeutic use of probiotics has increased due to their ability to modulate immune-inflammatory responses.^14–16,18,29^ In Dentistry, probiotic strains have shown efficacy in treating oral diseases.^21,30^ Among these strains, *Bifidobacterium animalis* subsp. *lactis* HN019 has demonstrated promising effects, particularly in Periodontology^20,31,32^ and Endodontics.^23,33^

The present study evaluated the effect of *Bifidobacterium animalis* subsp. *lactis* HN019 as a root canal irrigant on M1 and M2 macrophage polarization in experimentally induced periapical lesions in rats. The null hypothesis, which stated that the probiotic irrigation would not influence macrophage polarization or the immune-inflammatory response compared with conventional irrigation or no irrigation, was partially rejected. Although no significant differences were observed among the groups at the early and intermediate experimental periods, the probiotic solution significantly increased NOS2 immunostaining at 42 days compared to the non-irrigated group, suggesting a higher presence of M1 macrophages, which are associated with pro-inflammatory responses.^3,4,34^ However, no significant differences were found in CD163 immunostaining (M2 subtype) among the experimental groups across all periods.

In the context of periapical lesions, M1 polarization is linked to the initial and chronic phases of inflammation, while M2 macrophages play a role in inflammation resolution and tissue regeneration.^3,4,5,35^ In this study, since NOS2 immunostaining was significantly elevated at 42 days, it is suggested that the probiotic may prolong the initial inflammatory phase to support effective elimination of pathogenic microorganisms. A robust initial phase may benefit the resolution of resistant infections, a phenomenon previously observed in other studies using probiotics in antimicrobial therapies.^36^ As noted in the literature, the use of *Bifidobacterium animalis* subsp. *lactis* HN019 as an irrigant was able to modulate the immune- inflammatory response,^14–16,17,36^ suggesting that this could be an effective alternative compared to sodium hypochlorite, which exhibits cytotoxic effects on surrounding tissues.^37,38^ However, while probiotics are known for promoting an anti-inflammatory response,^5,6,39^ this effect was not as pronounced in periapical lesions over the experimental periods in this study. This may be related to several factors, such as treatment duration, the animal model used, or even the probiotic dose administered.

Some methodological aspects of the present experimental model should be considered when interpreting the findings. The use of a well-established rat model of apical periodontitis represents an important strength of this study, as it allows controlled induction and progression of periapical lesions under standardized biological conditions. In addition, the use of immunohistochemical analysis enabled the quantitative evaluation of macrophage-related markers in the apical and periapical regions, contributing to a more specific assessment of the immune-inflammatory response. However, some limitations should be acknowledged. Immunohistochemical analysis provides indirect evidence of macrophage polarization based on marker expression. Furthermore, the evaluation was limited to two markers (NOS2 and CD163), which may not fully capture the complexity of macrophage phenotypes. In addition, no structural or morphometric analyses, such as lesion size measurement, micro-CT evaluation, or bone resorption assessment, were performed, which limits the ability to correlate immunological findings with tissue repair or lesion progression.

Therefore, this study demonstrated that *Bifidobacterium animalis* subsp. *lactis* HN019 exhibits a modulating effect on inflammation, with a significant increase in M1 macrophages 42 days after pulp exposure. However, there was no significant induction of M2 macrophages, indicating the need for further studies, including clinical trials, to confirm the effects of probiotic solutions on macrophage polarization in root canal therapy.

## Conclusion

Based on the methodology used, it was concluded that the probiotic *Bifidobacterium animalis* subsp. *lactis* HN019 has potential for modulating the immune-inflammatory response in periapical lesions, as it increased NOS2 expression, a marker for M1 macrophages, over the course of apical periodontitis development. However, the probiotic-based solution did not alter CD163 expression, a marker for M2 macrophages.

## Data Availability

The datasets generated during and/or analyzed during the current study are available from the corresponding author on reasonable request.
